# Humanoid patient robot for diagnostic training in medical and psychiatric education 

**DOI:** 10.3389/frobt.2024.1424845

**Published:** 2024-10-09

**Authors:** Patricia Schwarz, Sandra Hellmers, Sebastian Spanknebel, Rene Hurlemann, Andreas Hein

**Affiliations:** ^1^ Assistance Systems and Medical Device Technology, Department for Health Services Research, School of Medicine and Health Sciences, Carl von Ossietzky University, Oldenburg, Germany; ^2^ Department of Psychiatry and Psychotherapy, School of Medicine and Health Sciences, Carl von Ossietzky University, Oldenburg, Germany

**Keywords:** human-robot interaction, robots for educational purposes, simulated robot patient, humanoid robot Ameca, patient robot for medical education

## Abstract

Simulation-based learning is an integral part of hands-on learning and is often done through role-playing games or patients simulated by professional actors. In this article, we present the use of a humanoid robot as a simulation patient for the presentation of disease symptoms in the setting of medical education. In a study, 12 participants watched both the patient simulation by the robotic patient and the video with the actor patient. We asked participants about their subjective impressions of the robotic patient simulation compared to the video with the human actor patient using a self-developed questionnaire. In addition, we used the Affinity for Technology Interaction Scale. The evaluation of the questionnaire provided insights into whether the robot was able to realistically represent the patient which features still need to be improved, and whether the robot patient simulation was accepted by the participants as a learning method. Sixty-seven percent of the participants indicated that they would use the robot as a training opportunity in addition to the videos with acting patients. The majority of participants indicated that they found it very beneficial to have the robot repeat the case studies at their own pace.

## 1 Introduction

Simulation-based learning is considered an essential part of practical learning. Medical simulation offers many potential strategies for comprehensive and practical training, and safer patient care. On the one hand, the refinement of technical skills is learned through simulation using the latest technology, and on the other hand, social interaction in the so-called doctor-patient conversation is also simulated and trained, see Pierre and Breuer (2013). Teaching communicative skills in medical education has become increasingly important in recent years. The simulation patient program is an established part of the medical studies curriculum. Communication skills are regarded as an important element of the medical consultation and make up part of patient-centered care, see for example, Marcinowicz and Górski (2016) and Kee et al. (2018). Some consultations are more challenging than others, such as presenting bad news to a patient or talking to an angry or unmotivated patient. Overall, patient contact enables medical students to have a holistic learning experience and promotes their interpersonal skills and professional identity. It is an essential part of medical education to develop a comprehensive understanding of patient care, see Lewis et al. (2017) and Kiluk et al. (2012). To simulate a patient in a learning scenario, there are a variety of options, such as role-playing games or patients simulated by professional actors, see Langewitz (2012). Professional actors as simulated patients (SPs) are currently used in medical teaching to realistically simulate situations Peets et al. (2010). The professional actors learn their role from a script and act out specific symptoms. By working with SPs in education, medical students are given the chance to improve their patient relationship competencies, such as using active listening techniques and empathic responses, in a safe and realistic way (see, for example, Subodh, 2018; Velásquez et al., 2022). However, professional actors require a high organizational and financial effort, which only allows the students very limited practice opportunities, Pierre and Breuer (2013). This prevents the students from being able to practice collecting the findings independently and of the course at their own learning pace. Besides using professional actors, humanoid robots can also be used to simulate disease-specific patient behavior and create realistic practice scenarios for students. The robot can simulate diseases reproducibly and is able to simulate human-like movements such as facial expressions and gestures, speech communication and thus emotions through appropriate programming. This allows students to develop diagnostic skills and practice a large number of case scenarios and different symptoms in variations at their own learning pace, since the robot offers high temporal availability. Due to technological progress, robots are becoming increasingly relevant in education and the healthcare sector in general. Robots in healthcare education have great potential for teaching by standardising processes, supporting learners independently and enabling collective learning. However, despite the existing benefits, further research is needed to integrate robots as teaching aids in healthcare education Marcos-Pablos and García-Peñalvo (2022). When talking about the social relevance of robots in the healthcare sector in general, the use of assistive technology in medicine and care is often cited as an example. Social (humanoid) robots, for example, are presented as a solution to the care crisis, although there are doubts about their technological maturity and there are no concrete application scenarios in everyday care, Maibaum et al. (2022). A systematic review found that people generally have positive attitudes towards social robots and are willing to interact with them. However, more research is needed to fully understand the factors that influence attitudes such as domain of application, robot design, as well as gender, age and cultural background of the users Naneva et al. (2020).

The aim of this article is to present a concept of a humanoid robot as a SP for medical education. Therefore we introduce the doctor-patient-conversation and its aspects as a future training scenario.

### 1.1 Scenario of a doctor-patient-conversation

**FIGURE 1:**
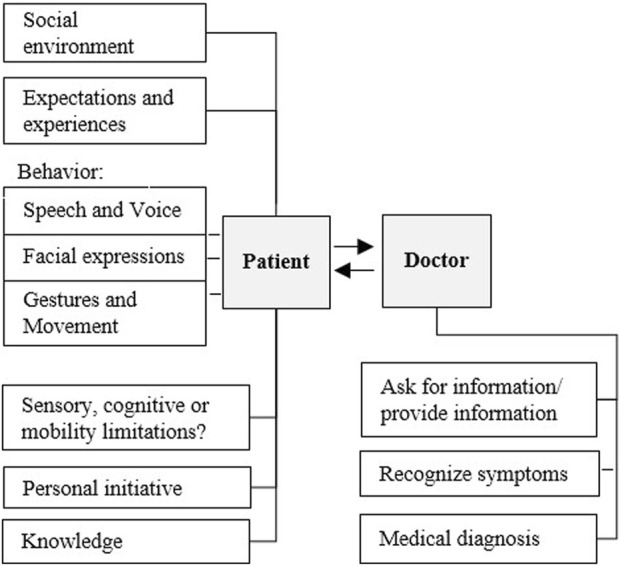
System diagram with different aspects of doctor-patient-conversation.

There are two different roles and perspectives - on the one hand the patient, who shows a certain disease-specific behavior - and on the other hand the doctor, who perceives this behavior, classifies it and makes a diagnosis based on it. Important features for disease-specific behavior of the patient can be recognized in his voice, gestures, facial expressions and his movements (especially in the case of psycho-pathological and neurological symptoms). In a learning setting of medical education, the role of the doctor is represented by the student. The role of the patient is represented by the actor (simulation patient). The Figure 1 shows a general model of the doctor-patient conversation. In our study, the simulation patient is replaced by the humanoid robot Ameca. The doctor takes the patient’s medical history and expects the patient to describe his or her condition and complaints. The doctor must interpret the sometimes unclear descriptions of the patient’s disturbed general condition, summarize them in a diagnosis and return them to the patient in a language that the patient can understand. The diagnosis forms the basis for therapeutic measures. Therapy, similar to medical history, is largely a communicative act. The doctor must explain to the patient the connections between complaints, the disease process and the medical therapy suggestions. The success of the therapy is all the more the better the therapeutic working alliance is. In order to be able to build this alliance, it is important to achieve the right linguistic access to the patient through communicative work.

To train the recognition of complex diseases, we are developing a learning scenario with a simulated patient. To simulate a patient, different possibilities are available:

• Real patient contact

• Humanoid robot as simulated patient (physical presence)

• Traditional education: Professional actors as SPs (physical presence)

• Educational videos of professional actors as SPs (digital education)

• Dynamic patient simulation via VR simulation (digital education)

• Digital virtual patient agents (digital education)

**FIGURE 2:**
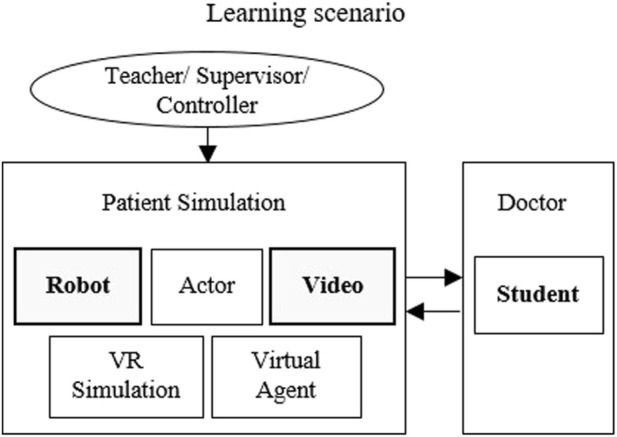
Possible patient simulation options.

Figure 2 shows the possibilities of patient simulation in the learning scenario, as well as the two simulation methods we chose for patient simulation. In a learning scenario, there is also the role of the lecturer, teacher or supervisor, who instructs the simulation patient and determines the scenario. When using the robot patient, these instructions are transferred to the robot by a controller. The virtual/robotic simulation patients are based on a behavioral model, which is developed and accessed together with lecturers, developer and robot controller.

We chose to compare the humanoid robot SPs with instructional videos of an professional acting patient in our study. The videos provide a degree of reproducible that would not be available when compared to an human actor as SP.

## 2 Related work

The aim of this article is to present a concept of a humanoid robot as a SP for medical education. Therefore, we will first explain which types of human-like robots have already been developed and why a humanoid robot represents a practical solution to support learning processes in medical education and training.

First human-like communication robots with a very human-like appearance have already been developed for medical training in the past. Hashimoto et al. (2011) developed the Android robot “SAYA,” which communicates emotionally with people as an interactive communication system. Since the face and its facial expressions play the most important role in natural communication, Saya can make facial expressions similar to humans. SAYA has been used in studies as patient with depression to provide reproducible and realistic education and training for prospective psychiatrists. In particular, the patient robot treats unipolar depression, a typical psychiatric illness. The robot shows symptoms of unipolar depression by exploiting its communicative functions such as facial expressions, eye and head movements, and distinctive language. During an interview with a psychiatrist, the robot’s communication functions are controlled remotely and the interview proceeds according to a previously prepared diagnostic scenario, see Hashimoto et al. (2006).

Röhl et al. (2023) developed an android robot-patient (ARP) to train non-verbal communication with critically ill intensive care dependent patients. Individual behavior patterns of real patients were observed using a multi-sensor system and transferred to the ARP. In order to provide a teaching, training and assessment method for larger groups of students, an ARP has been developed that simulates different types of delirium. Another example is the Patient Robot from Tanzawa et al. (2012), who developed a robotic patient for dental clinical education that can accurately reproduce authentic clinical situations. The robot patient has been improved so that many trainees can use it permanently. The rationale behind the development of the robotic patient included presenting a full body and reproducing autonomous movement through robotics, as well as enabling a conversation with the trainee, see Madokoro et al. (2007). Very few previous works specifically consider the impacts of humanoid robots within a educational context. According to studies by Okita et al. (2009) and Dautenhahn (2007), students prefer robots that have human-like behavior, appearance and a human-like voice. Although the use of simulation robots in medical education has a positive impact on experimental learning performance, most currently commercially available simulation robots lack realistic facial expressions and social features. A study by Martin et al. (2012) suggests that this lack of facial expressions can lead to poorer transmission of skills and eventually to adverse outcomes for the patient. Another challenge of the currently available simulation robots is their control and usability.

Moosaei et al. (2017) show that these are difficult for educators and teachers to control - especially when more complex simulations are run. It is therefore important to include the working methods of teachers during the simulations in order to best support changes in their workflow and to create a common control system. We have already carried out a pre-study as part of our own preparations (Schwarz and Hein, 2023). The aim of the study was to investigate whether the humanoid robot is able to reproduce patient behavior realistically. In particular, we examined the robot’s technical functions and characteristics. The pre-study served as preparation for the feasibility study described here. A review of the literature shows that the idea of using humanoid robots in teaching is not entirely new, but its use in medical education for the purpose of doctor-patient communication is very rare. In this article, therefore, a humanoid robot is used for this purpose, which offers a broader range of expression and control capabilities compared to other commercially available systems. Studies that examine modern humanoid robots as simulated patients in doctor-patient conversations or that test different simulation methods against each other are rare.

## 3 Methods

### 3.1 Comparison of patient simulation methods

**FIGURE 3:**
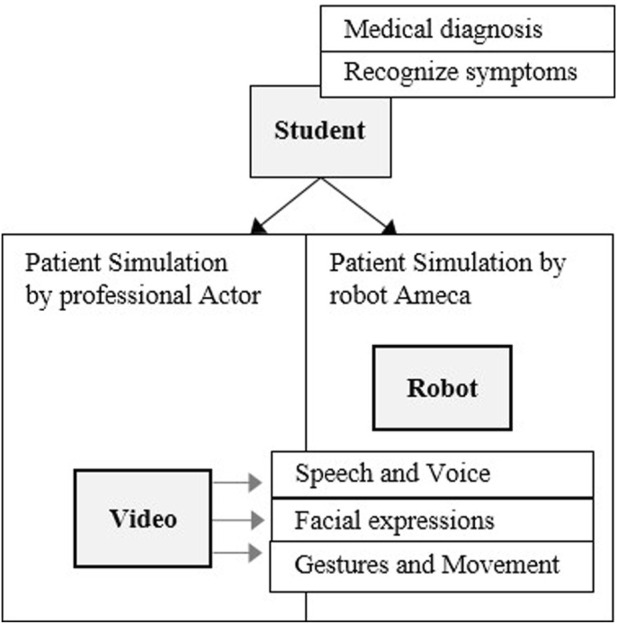
Simplified diagnosis situation - patient simulation by humanoid robot, compered to patient simulation by video.

In our study we investigate the use of a humanoid robot as a simulation patient (Figure 3). The representations of the robot patient are limited to speech, facial expressions, gestures and movements. The robot patient plays back predefined sequences in which it represents a symptom of the disease. The student (in the role of the doctor) watches at the simulated sequence and identify the symptom. We compare two simulation methods by participants rating, so videos with professional patient actors are more suitable, because a human would not be able to reproduce the simulated sequence identically over and over again and thus there is no direct comparison possibility to the robot simulation.

### 3.2 Research questions

With this work, we broadly address four research questions (RQ). One aim of our study was to find out which subjective differences the participants perceive when the shown disease symptoms are represented by an professional patient actor in the video or by the patient simulation robot. For example, disturbing characteristics will be identified in order to optimize the robot simulation. Subjective questions allow participants to express their personal opinions, experiences, or perceptions about the robot simulation and the actor-patient video. Responses to subjective questions are intended to help us understand a person’s point of view or attitude and to identify possible patterns, trends, or differences in participants’ opinions.

Furthermore, we want to investigate and evaluate to what extent the patient behavior from the videos can be simulated as realistically as possible with the robot under the technical conditions with the appropriate software. In contrast to subjective questions, which target individual perspectives and feelings, an objective comparison of simulations using motion tracking is intended to focus on concrete facts and observable information. In addition, we would also like to investigate to what extent the humanoid robot is accepted by the participants as a simulated patient in the representation of symptoms as a training option. For this purpose, a standardized questionnaire will also be used to record a person’s tendency to actively engage with technological systems.

RQ1a: What are the differences between watching patient simulation videos by the actor and experiencing patient simulation by the robot Ameca when presenting symptoms (subjective comparison)?
RQ1b: What features and functions do subjects find most useful and realistic?
RQ2: To what extent can the patient behavior shown be realistically transferred to the robot (objective comparison)?
RQ3: Is there some acceptance of the humanoid robot as the simulation patient compared to the videos with the professional actor patient?
RQ4: How does a person’s affinity for technology affect their willingness to interact with robots?

Hereby the respective characteristics, the acceptance and the difference perception between robot and video presentation are dealt with by an appropriate questionnaire. Parallel to this, an objective comparison using motion tracking software will measure whether differences in the respective patient presentations (movement, speed) can be determined. From the findings of both studies (subjective and objective comparison) we want to deduce whether the robot was able to simulate the patient behavior realistically.

**FIGURE 4:**
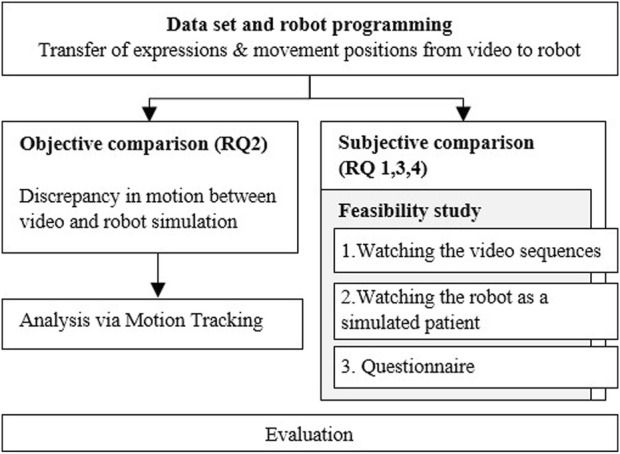
Process diagram of the study design.

The process diagram in Figure 4 shows the individual steps. In our study we examine and compare two simulation methods of patient representation, on the one hand by videos with acting patients - on the other hand by a humanoid robot - for the purpose of training and practicing the assessment of findings in medical education.

Videos from the medical teaching of the Clinical Training Center (2023) (KTZ) of the University of Oldenburg were made available to us as a basis for the robot simulation. In these videos, an professional actor presents psychopathological symptoms. In a next step, the representations of the actor, such as facial expressions, language and movements, were simulated and transmitted as parameters in the robot software. Finally, the virtual simulation was transferred to the humanoid robot, which recreated the symptoms shown in the video. In order to compare the simulation methods of the patient representation in the video and the patient representation via the robot, a feasibility study was carried out. The selection of participants for the feasibility study is discussed in Section “Participants.” The participants watched both the video and the robot simulation and then completed a questionnaire for comparative evaluation. The questionnaires are then evaluated. Additionally to the subjective statements of the participants, we do an objective measurement of differences between video and robot representation.

## 4 Study design

Our intention is to simulate an interaction between doctor and patient with our robotic simulation patient. In this study, the simulation patients presented symptoms that were observed by the participants in a passive presentation. There was no interaction between the simulation patient and the participant at this point.

### 4.1 Study procedure

The procedure was explained to the participants at the beginning and the aim of the study was presented.

In the first step, the participants watched three videosin a row with depictions of three different psychopathological symptoms by an professional actor. The correspondingly presented psychopathological symptom was announced to the participant in advance. In this study, the participants should focus on the simulation method (video vs. robot), not primarily on identifying the displayed symptom.

In our comparison, a passive presentation of disease symptoms by a robotic simulation patient and a video of an acting patient takes place. There is no interaction between the participant and the robot.

The video sequences each have a duration of 0.3–3 min and each show a presentation of a finding by the patient actor. Watching the three videos takes a maximum of 10 min. Psychopathological findings presented include shifts in consciousness, depersonalizing, and affect liability.

Then the participants were asked to look at the same psychopathological findings - this time simulated by the robot. The robot reproduced the actor’s patient behavior in the videos. The presentation of the findings by the robot take about 10 min.

### 4.2 Data set and robot

#### 4.2.1 Robot

Engineered Arts Ltd. (2022) designed Ameca to resemble the human body. The robot has a human-like anatomy with arms, legs, a head and a face.

With the ability to show smiles, eye movements, head nods and other human expressions, the robot can convey emotions and intentions and create a deeper connection with humans. Also, the ability to speak and have a human-like voice allows Ameca to communicate in a way that is understandable and familiar to humans.

Ameca’s behavior can transferred by using the simulation program “Virtual Robotics.” Virtual Robotics provide virtual environments for the simulation and development of robots, so users can simulate the behavior of robots and create robot models, program their movement and simulate different scenarios. The 3D robot moves and talks exactly like the real robot will.

**FIGURE 5:**
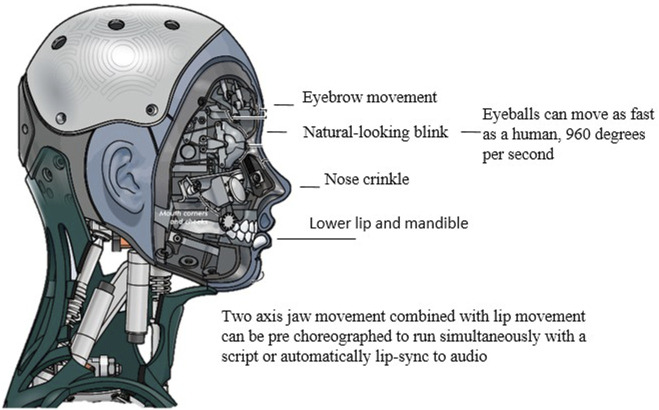
Technical details of robot Ameca, Engineered Arts Ltd. (2023).

The robot’s face is covered with a flexible coating that can be deformed in a variety of ways, Figure 5.

#### 4.2.2 Data set

**FIGURE 6:**
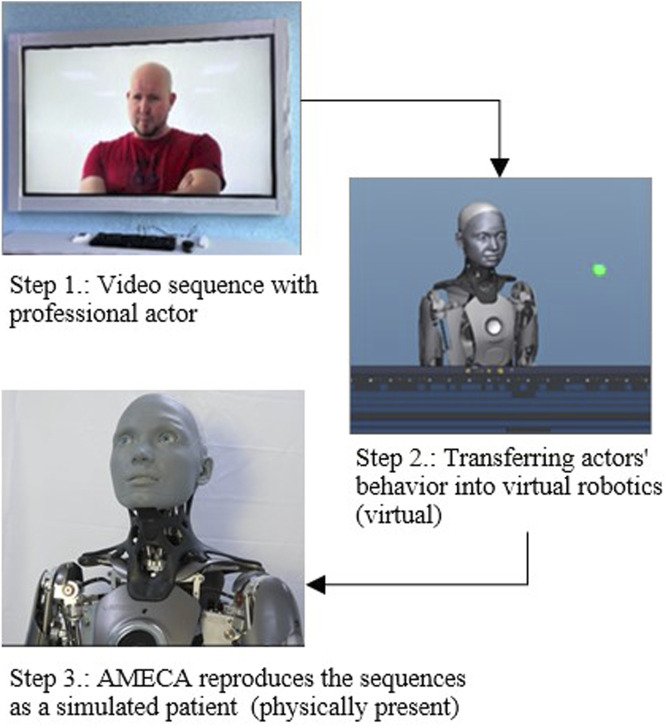
Transfer of the behavior of the actor in the video to the robot Ameca via the simulation program “Virtual Robotics”.

The behavior of the actor in the video was transferred using the simulation program “Virtual Robotics” to Ameca. By using Virtual Robotics, users can simulate the behavior of robots in virtual environments, create robot models, program their movement and simulate different scenarios to test and optimize the robots’ behavior. As a result, both facial expressions and movement positions of the acting patient can be transferred to the humanoid robot using various parameters. The actor’s video recording thus provides the basis for simulating the behavior of the robot in the virtual environment, Figure 6. If the facial expressions or movements of the actor in the video were changed accordingly, these parameters were adjusted accordingly in the robot simulation until the sequence matched to a certain extent. This conversion and transfer of the movements from the video to the robot was done manually for all three psychopathological symptoms.

### 4.3 Video sequences

**FIGURE 7:**
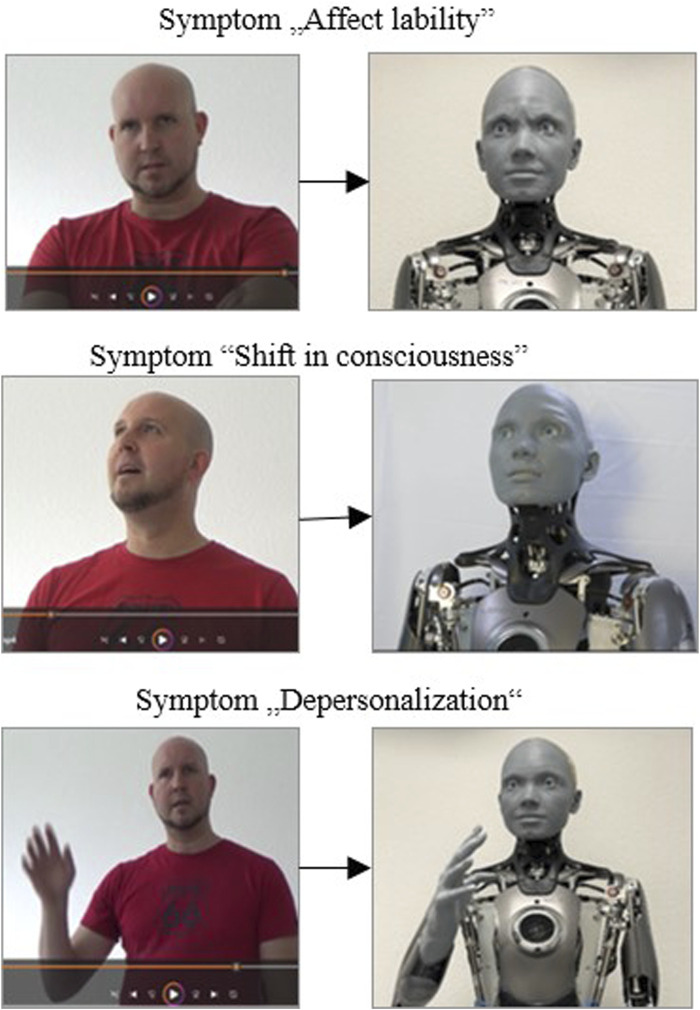
Presentation of the psychopathological symptom by the actor in the video vs. presentation by Ameca as a simulated patient.

The following figure (Figure 7) shows a representation of the psychopathological symptom by the actor in the video in direct comparison to the representation by Ameca as a simulated patient. The symptoms presented are briefly explained below.

Affect-lability is a short-term, abrupt alternation of emotional expressions with a rapid change (e.g., from joyful excitement to tearfulness even at the slightest occasion), Figure 7, upper picture. Shift in Consciousness (middle picture)- This is a shift in consciousness associated with a feeling of increased intensity and brightness in terms of alertness, perception of interpersonal or external processes. Perception appears more vivid and more emotional. In the depiction of the psychopathological symptom of depersonalizing (Figure 7 lower picture), the actor portrays a person who experiences himself as alien and changed. With these symptoms, one’s own reactions and sensations, but also one’s own body is experienced as changed and unfamiliar, or something foreign is perceived in it.

### 4.4 Participants

We recruited 12 participants (11 medical-, psychological- and applied nursing science students and one nurse) for the study, Table 1 provides additional demographic details. The participants were recruited by personal contact at the University of Oldenburg (D). The participants were between the ages of 21 and 50, 9 women and 3 men. Eight participants have professional experience in a medical context, between 1.5 and 30 years. For the age of our sample size of n = 12 test participants, we calculated a mean of m = 27.83 years and a median of x = 25 years. The first quartile corresponds to 24 years and the third quartile to 28 years.

**TABLE 1:** Participants n = 12, G, Gender; Med, Medicine; Psy, Psychology; ANS, Applied nursing sciences; N, Nurse.

Age	3 male, 9 female	Degree	Work experience available	Work experience (years)
24	f	Med	Yes	1, 5
24	m	Med	Yes	4
26	m	Psy	No	0
21	f	Med	No	0
24	f	Psy	No	0
25	f	Med	Yes	8
25	f	Med	Yes	6
24	m	Med	No	0
31	f	ANS	Yes	6
27	f	ANS	Yes	8
25	f	ANS	Yes	5, 5
50	f	N	Yes	30

The following inclusion criteria were used:

• Students from the fields of medicine, psychology, applied nursing science and nurses

• Age: older than 18 years

Exclusion criteria:

• No relevant reference to the setting of the study

• Inability to understand the course content and course of study

• Severe visual limitations that do not allow the exercises to be carried out

The study was approved by the medical ethics committee of the University of Oldenburg (05/08/2023 - vote 2023-086). Written informed consent was signed prior to the study.

### 4.5 Questionnaires

After the two simulation methods (video presentation with an actor patient and a humanoid robot as a SP), the participants filled out a structured questionnaire as a comparative evaluation. The questionnaire consists of three sections: General questions, questions on interaction-related technology affinity (ATI-Scale by Franke et al. (2019)) and questions on difference perception related to video and robot. We have chosen our own questionnaire for the subjective comparison between humanoid robots and video with actors, as standardized instruments did not seem suitable here. The reason is that the participants do not use the robot for anything or do anything with it, but simply observe the robot as it describes its symptoms as a simulated patient.

The ATI-Scale offers a quick method to map the technology affinity of the participants on a dimension from very little tech affinity to very high tech affinity.

For this purpose, the participants were presented with nine statements to which they were asked to respond for themselves on a six-point response scale from “not true at all” to “completely true.” The ATI questionnaire was used to measure the participants’ general attitude towards technology or robots.

Here, for example, questions are asked about how realistically the robot has represented the patient (optical/acoustic) or what needs to be changed in the representation so that the robot appears more realistic as a patient actor. Responses were given on a 5-point Likert scale of Strongly Agree (1), Rather Agree (2), Rather Disagree (3), Disagree (4), and Strongly Disagree (5).

In addition, the participants were asked whether, in their opinion, the robot as a SP would offer a useful addition to independent learning as an additional exercise option. The subjective evaluations of the participants as free answer options in the questionnaire also serve to answer the question of which functions and properties appear to be particularly important to the participants. The evaluation of the study should also show to what extent the patient behavior shown can be realistically transferred to the robot.

### 4.6 Objective difference measurement between video and robot representation

To verify the subjective impressions of the participants objectively, the movements in the faces of the professional actor and the robot were recorded and compared using the motion tracking software “Adobe After Effects.” The basis for this was an identical 10 s long video sequence by the actor and by Ameca.

**FIGURE 8:**
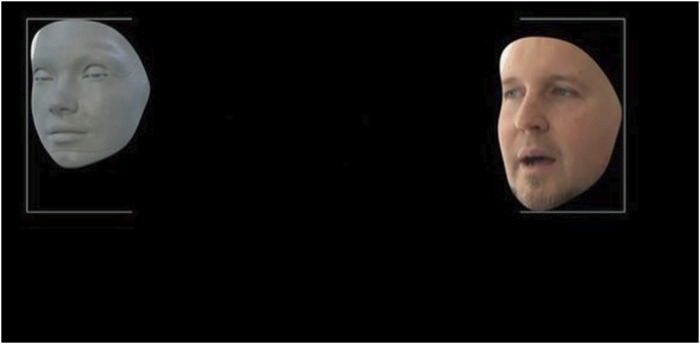
Motion tracking: comparison of facial movements of professional actor and robot Ameca.

**FIGURE 9:**
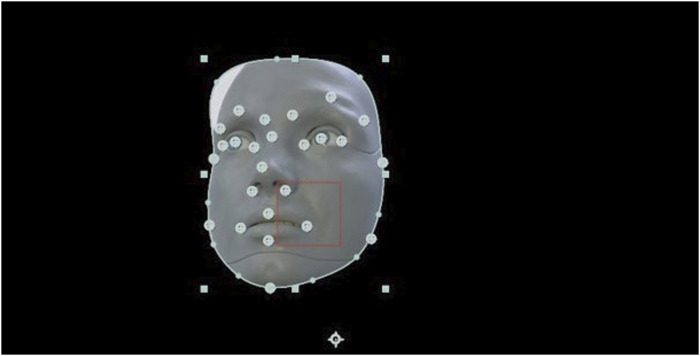
After effects tracks motion of Amecas face.

Motion tracking allows the movement of an object to be tracked and the tracking data for that movement to be extracted (Figure 8). The software tracks motion by matching image data from a selected area in one frame to image data in each subsequent frame. The area to trace was specified by setting trace points in the Layers panel (Figure 9). Each track point contains a feature region, a search region, and a connection point.

## 5 Results

In the following sections we describe the results of our study.

Unlike the objective questions, which aim at concrete facts or observable information, subjective questions refer to individual perspectives and feelings. This part of the questionnaire, developed by us, is intended to collect qualitative data and to gain insights into the thoughts, opinions and preferences of the participant. The answers to subjective questions are designed to help us understand a person’s point of view or attitude and to identify possible patterns, trends, or differences in participants’ opinions.

### 5.1 Subjective comparison

Through the subjective comparison, we wanted to find out how the participants experience the differences between watching videos with patient simulation by an professional actor and experiencing the patient simulation by the Ameca robot when presenting symptoms. In addition, it should be evaluated which properties and functions the participants find particularly useful and realistic. In this part, questions were asked aimed at direct comparison of the simulation method (educational Video vs. Robotpatient).

Q1 How realistically are patients portrayed by the actors in the video?
Q4 For me, the physical presence of a human-like robot represents added value compared to a video.
Q5 The robot is a useful addition as an additional exercise opportunity and promotes independent learning.
Q6 I believe that training with robots offers a useful and complementary learning method in my medical training.
Q7 I see it as a great advantage to have the case studies repeated to me by the robot at my own learning pace.
Q9a Are patients realistically visualized represented by the robot?
Q9b Are patients realistically represented acoustically by the robot?

**FIGURE 10:**
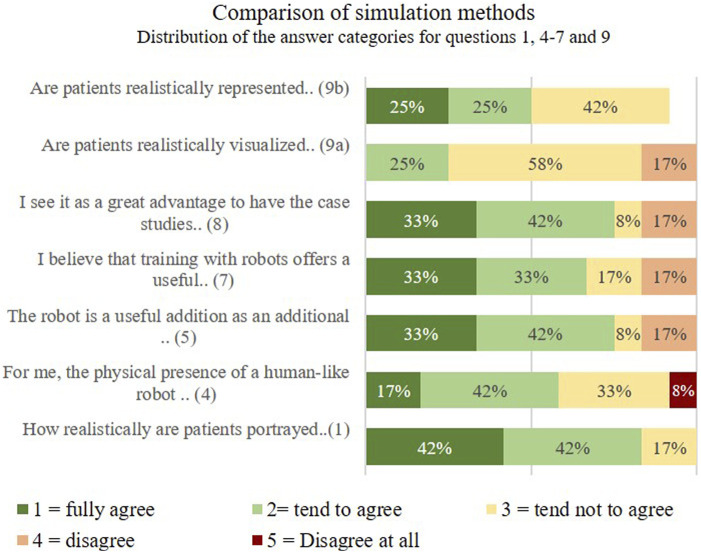
Comparison of simulation methods.

Figure 10 shows the distribution of the answer categories for questions 1, 4–7 and 9 for the individual scale items.

The participants watched both the video and the robot simulation and then completed a questionnaire for comparative evaluation. In question 1, whether patients are portrayed realistically by the actors in the video, 42% of the participants fully agree, 42% tend to agree. 17% of those surveyed tend not to agree that the actor plays the patient realistically. In question 4, the participants were asked to judge whether the physical presence of a human-like robot represented added value for them compared to a video. Here 17% and 42% fully agreed/somewhat agreed, Figure 10.

Question 5 “The robot is a useful supplement as an additional exercise opportunity and promotes independent learning” was answered with 33% fully agree, 42% tend to agree and 25% disagree.

For question 6 “I believe that training with robots offers a useful and complementary learning method during my training as a doctor.” 33% of those surveyed fully agree, and 33% also tend to agree. In question 7, the participants were asked whether they saw it as a great advantage to have the case studies repeated by the robot and at their own learning pace. Here the majority fully/rather agreed. The question of whether realistically patients are visually represented by the robot was answered with 58% of those questioned with disagree. 25% of respondents tended to agree. 50% tend to agree and 42% tend to disagree as to whether patients are represented acoustically realistically by the robot.

### 5.2 Objective comparison

In an objective comparison, we would like to verify the feasibility of realistically transferring the demonstrated patient behavior to the robot. To do this, we use objective comparison through the use of motion tracking. We placed something like 20 different tracking points on the face of the actor and the robot and tracked them over a video sequence with a length of 10 s. This resulted in about 250 x and y coordinate points for each tracking position.

We have graphically displayed the course of three selected tracking points as examples to graphically show the differences in the motion sequences between actor and robot.

The tracker determines the position where the search area and reference pattern match most accurately. For each analyzed image, the tracker determines an assignment value (x and y value in the pixel coordinate system).

**FIGURE 11:**
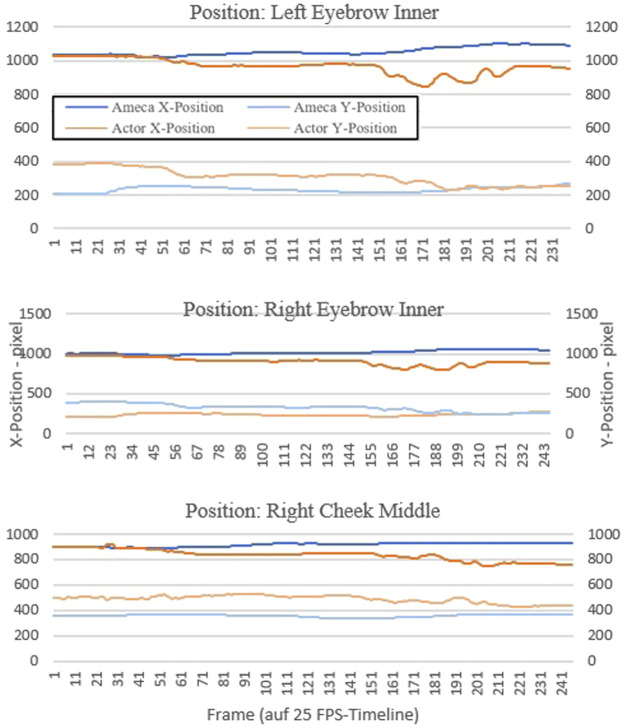
Results motion tracking of the video sequences “affect lability”.

**FIGURE 12:**
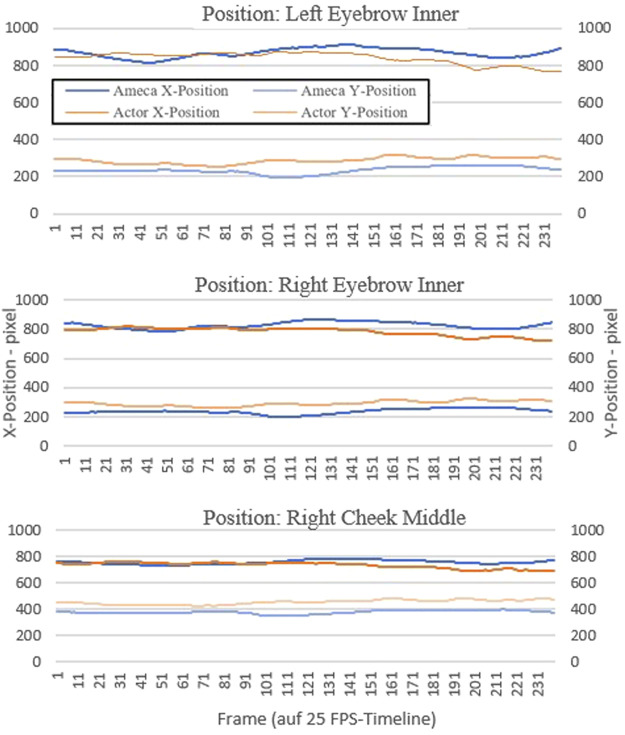
Results motion tracking of the video sequences “consciousness shift”.

For this purpose, we chose the tracking point of the left and right inner eyebrow, as well as the right middle cheek. These three points represent the motion changes in the face better than other areas in the face, such as the forehead or the mouth area. The diagrams in Figures 11, 12 shows the progression of each tracking position over time, with the robot’s movement shown in blue and the actor’s movements in orange. Robot and actor play an identical sequence.

### 5.3 Acceptance of the humanoid robot as simulation patient

It should be investigated whether there is a certain acceptance of the humanoid robot as a simulation patient compared to the videos with the professional actor patient. The questions are about the simulation method and for the purpose of further developing the robot:

• Question 2: What was realistically rendered by the actors in the video?

• Question 3: What should be changed about the representation in the video so that the actor appears more realistic?

• Question 8: Did you feel that the robot was different from a real patient? If so, what are the differences?

• Question 10: What was realistically rendered by the robot?

• Question 11: What should be changed in the display to make the robot look more realistic?

We questioned the participants, which was realistically designed by the actors in the video. When asked what was realistically portrayed by the actor in the video (Q2), most respondents pointed out that gestures and facial expressions were very clear, so that emotions become clear even without the content of the conversation (12 participants’ statements). Physical reactions such as rapid breathing and movements were also mentioned (1 participant). Another participant noted that the actor’s eye contact, intonation and voice pitch were also listed as realistic criteria. During the simulation of the “Shifting Consciousness” sequence, noise was said to have been detected in the sound (1 participant).

In response to the question 3 “What should be changed about the video presentation to make the actor appear more realistic?,” one respondent points out that a more suitable environment - sitting with furniture or a room in the background - could have a positive effect. Another participant noted that the setting should take place in a practice setting. Another respondent here mentions that the simulated case presentations should show comorbidities. One other participant noted that the emphasis on the language seems posed. In question 8, participants were asked if they felt the robot was different from a real patient. If the answer was yes, they should name the differences. One view was that emotions and gestures were not as diverse, especially euphoric emotions. Several respondents indicated that speech and lip movements were not always in sync (3 participants’ statements). Another respondent indicated that the facial expressions were not as flexible and there was a lack of eye contact from the robot. In addition, the facial expressions were not perceived as being as fine as in the video, the robot’s face seems rather slowed down, especially with more aggressive language. One participant remarked that the robot’s movements were too slow. Another participant stated that the optics and facial expressions are not yet 100% realistic, but are surprisingly similar. The respondents of two participants stated that the movements of the robot were sometimes too jerky.

When asked what was rendered realistically by the robot in question 10, two respondents indicated that depressed, calmer mood, upper extremity gestures and the face in general were rendered very realistically. Another participant noted here that acoustics and lip syncing were perceived as very realistic. The eye movements were also realistically represented by the robot. Furthermore, one respondent remarked that the language was represented very realistically by using the original soundtrack (4 participants’ statements). Another participant indicated that the robot was intimidating by maintaining eye contact. It was also noted that the robot’s movement executions were a bit too smooth.

Finally, the participants were asked what should be changed in the display so that the robot appears more realistic (question 11). One perspective was to put clothes or a wig on the robot if necessary and to avoid technical noises (2 participants). Three respondents indicate that facial expressions could be further improved. If possible, the speed of movement should be better adapted to the speed of speech. The lip sync could also be improved. It was also noted that lip reading was not possible (2 participants’ statements) and the robot lacked a tongue. This also makes it impossible for one participant to chew on the lips. Another respondent indicated that there was more interaction between the robot and the doctor.

In the questionnaire section “General Questions 1–5,” the participants were specifically asked in which form they would prefer to have the case studies presented. The results are shown below.

• Question 1: The simulation of case studies is an indispensable training method for me

• Question 2: I feel well prepared for doctor-patient communication as part of my studies

• Question 3: I understood the content of the case study presented and was able to classify it

• Question 4: After this exercise, I recognize my own difficulties in interaction communication and in the assessment of sick patients

• Question 5: In which form would you prefer to have the case studies presented?

a: In regular course dates with acting patients
b: In addition to the acting patient videos, I would use the robot as a practice opportunity
c: Through the videos
d: From Just with the Robot

**FIGURE 13:**
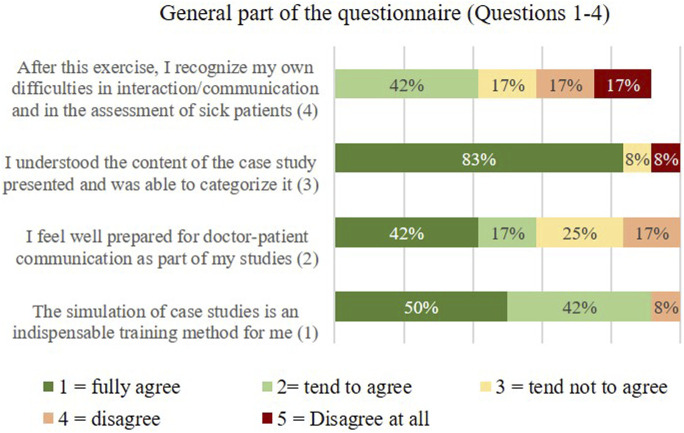
General part 1 of the questionnaire, Q1–4.

The first question “For me, the simulation of case studies is an indispensable exercise method” answered the overwhelming majority of the participants with yes (50% completely agree, 42% tend to agree), Figure 13. When asked whether the participant felt well prepared for doctor-patient communication during their studies (question 2), the majority tended to disagree (42% fully agree, 17 tend to agree). 25% and 17% respectively disagree rather disagree. Question 3 was used to evaluate whether the participants were able to understand and classify the content of the case studies presented (video and robot). (“I understood the content of the presented case study and was able to classify it”).

The results in Figure 13 shows, that 83% of the participants answered yes to this question. The 4th question served to record the self-assessment of the participants in the interaction and in the diagnosis of sick patients (“After this exercise I recognize my own difficulties in interaction/communication and in the diagnosis of diseased patients”). Here the answers were very different, which was to be expected from the heterogeneous group of participants.

### 5.4 Affect of a person’s affinity for technology

We wanted to find out how a person’s affinity for technology affects their willingness to interact with robots (RQ4). To this end, we used a standardized questionnaire. The following list shows the questions from the Affinity for Technology Interaction (ATI) Scale (Franke et al., 2017).

ATI1 I like to occupy myself in greater detail with technical systems.
ATI2 I like testing the functions of new technical systems.
ATI3 predominantly deal with technical systems because I have to.
ATI4 When I have a new technical system in front of me, I try it out intensively.
ATI5 I enjoy spending time becoming acquainted with a new technical system.
ATI6 It is enough for me that a technical system works; I don’t care how or why.
ATI7 I try to understand how a technical system exactly works.
ATI8 It is enough for me to know the basic functions of a technical system.
ATI9 I try to make full use of the capabilities of a technical system.

**FIGURE 14:**
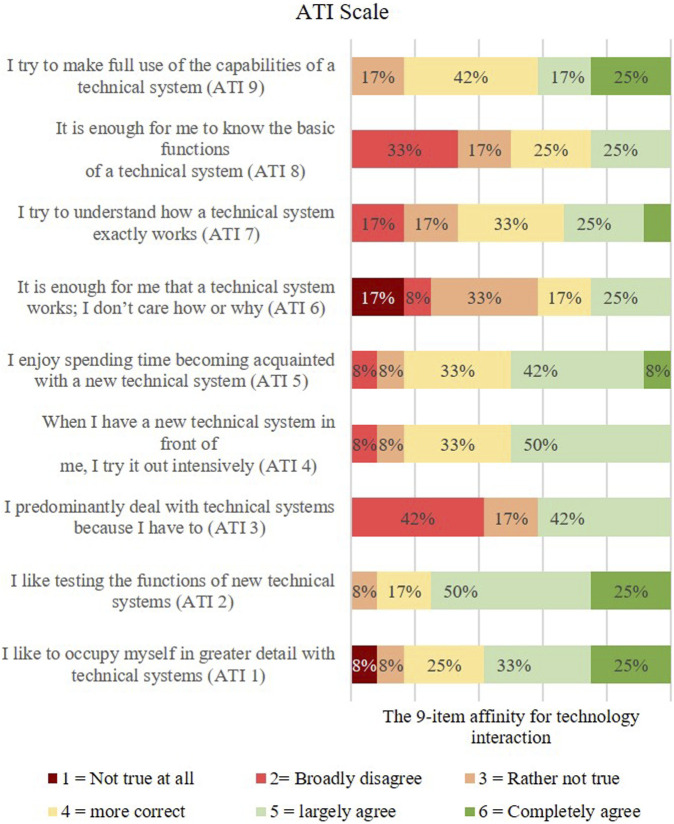
The 9-item affinity for technology interaction (ATI Scale).

The ATI uses a Likert scale with the 6 answer options coded as follows: Strongly disagree = 1, Strongly disagree = 2, Rather disagree = 3, Rather agree = 4, Strongly agree = 5, Strongly agree = 6. Looking at the ATI distribution, participants surveyed are open to technology and enjoy interacting with it. This could be explained by the age of the participants (M = 27, 2). Figure 14 shows the results of the technology affinity of our participants from strongly disagree (red) to strongly agree (green).

## 6 Discussion

The first research question was to investigate in a subjective comparison what the differences are between watching patient simulation videos with an actor and the experience of patient simulation by the robot Ameca when presenting symptoms. The majority of participants agree that the physical presence of a human-like robot represents added value for them compared to a video. For the majority of the participants, the robot represents a useful addition as an additional exercise opportunity and promotes independent learning (33% fully agree, 42% tend to agree). The question of whether training with robots was a useful and complementary learning method during my training to become a doctor was assessed differently. 33% of respondents completely agree, 33% somewhat agree. However, the majority of those participants said that they saw it as a great advantage to have the case studies repeated by the robot and at their own learning pace.

In our second research question, we wanted to use an objective comparison to determine the extent to which patient behavior can be realistically transferred to the robot. In a objective comparison, using motion tracking in video recordings, it was evaluated whether the robot actually seemed slower and less alive during the simulation. In the results of the motion tracking evaluation of the videos showing the symptom “affect lability” (Figure 11), it is noticeable that the movements in the faces of the actor and the Ameca robot differ more than in the first sequence “shift in consciousness” (Figure 12). The robot seems to be able to represent the facial expressions and gestures presented here less realistically than in the presentation of the first symptom. This result corresponds to the subjective statements of the participants, who experienced the robot as slower than humans when it came to displaying anger and aggression.

The third research question was to investigate whether there is a certain acceptance of the humanoid robot as a simulation patient compared to the videos with the professional actor patient. The majority of participants answered the question “In which form would you prefer to have the case studies presented?” by saying that they would use the robot in addition to the videos as a practice opportunity for the presentation of case studies (67%). The other part (33%) indicated that they would learn the case studies in regular course appointments with actors. None of the participants stated that they only watched the case studies to learn from the videos. From the possible answers given, it can be concluded that learning through interaction and experience with actors or simulation robots seems to be more effective than just watching videos. Learning with actors or robotic simulation patients requires active participation by the learner. By interacting with other people, information can be better processed and retained. The learner is actively involved, which can lead to deeper understanding and better memory.

Furthermore, a real learning setting with actors or robots enables the practical application of knowledge and skills, by applying what has been learned in real or simulated situations, the learner can develop problem-solving skills, gain practical experience and build competencies in a specific area.

The fourth research question aimed to investigate how a person’s affinity for technology affects their willingness to interact with robots. The evaluation shows that the participants’ affinity for technology is very high. A person’s affinity for technology can have an impact on their willingness to interact with robots, but this is not the sole determining factor. People who have a high affinity for technology and are open to new technological developments are more likely to be willing to interact with robots, see Franke et al. (2017).

However, it is important to note that willingness to interact with robots does not solely depend on tech affinity. Other factors such as personal experiences, individual attitudes, cultural and societal norms, and the specific application or task for which the robot is being used may also play a role. In addition, willingness to interact with robots may vary over time as technology advances and public perception and acceptance of robots may change.

## 7 Limitations

Our study is not without limitations. Due to the fact that the demonstration of the case studies only lasted up to 3 min each, the subjects may not have had enough time to get a comprehensive picture of the robot’s capabilities or to put themselves in the situation of the scenes presented. Furthermore, we only examined subjects’ intention to use humanoid robots versus videos for learning purposes (as a simulation patient in medical education) and their predictors. Therefore, it cannot be determined whether the use of humanoid robots leads to an increase in knowledge or skills. The subjects’ perceptions of what is more conducive to learning may differ from the research results.

In this feasibility study, the main focus was on the technical implementation and the ability of the humanoid robot. The robot merely simulated the behavior of a sick person - there was no interaction between the participant and the robot.

The case studies (depictions of psychopathological symptoms) examined in our study are narrow in scope and their full complexity is not covered. A short excerpt of the symptom presentation was played back via video and robot. Furthermore, we cannot tell how humanoid robots will perform compared to other forms of technology-based assistance. Evidence of this would be needed to assess whether the additional costs incurred by a humanoid robot (e.g., compared to a virtual agent) are justified. Unfortunately, it was very difficult to find enough participants from the fields of medicine, psychology and nursing. We therefore had to accept differences in the sample distribution with regard to professional groups, age and professional experience. In this feasibility study, we deliberately only used descriptive statistics, as the sample size is unfortunately too small and the results would therefore be inaccurate and unrepresentative.

## 8 Conclusion and future work

A total of 67% of the participants stated that they intended to use the humanoid robot as an opportunity to practice patient simulation in addition to the videos with actor patients. 33% of the participants prefer regular course dates with actors. Further investigations could aim to investigate which learning and support conditions are important for students and specialists in the medical field in order to better predict and, if necessary, specify the actual use of the humanoid robot. Considering the background of the participants or providing individual support could be important. An assessment-oriented learning environment also requires a high degree of adaptability of the robot and the learning system. A community-oriented learning environment in particular is an important element in which learners support each other. In this sense, the social presence of the robot could play an important role. How humanoid robots and humans can effectively work together to create a community-centric learning environment could be a promising approach for future research.

The aim of this study was to examine in a direct comparison what differences between watching the videos and experiencing the robot in the role of the patient in simple test situations (short sequence of a presentation of a psychopathological finding) can be determined. A humanoid robot Ameca from Engineering Arts was at our disposal for this. Furthermore, it should be examined to what extent the patient behavior can be realistically transferred to the robot and whether the participants show a certain acceptance of the robot for learning purposes. The robot as an additional exercise option was a useful addition for the majority of the participants, which promotes independent learning. Most respondents also saw it as a great advantage to have the case studies repeated by the robot and at their own learning pace.

The generalization of the results from the comparison between robot and video with actors as simulated patients requires differentiated approaches. The objective comparison using motion tracking showed that the robot was actually slower and less lively during the simulation. In the results of the motion tracking evaluation of the videos showing the symptom “affect lability,” it is noticeable that the movements in the faces of the actor and the Ameca robot differ more than in other sequences. The robot is therefore slower than the human when it comes to displaying anger and aggression. It should be taken into account that the robot has certain limitations in terms of emotional facial expressions or the ability to simulate complex human signals. This result from our specific environment could also be relevant for other situations and applications of this humanoid robot. Overall, the results of this study could also be interesting for other medical fields. Humanoid robots in physical presence also offer a number of advantages for social and cognitive scientists, which have an impact on their research and experiments. The humanoid robot offers the ability to precisely control and reproducibly reproduce behaviors, movements, facial expressions and spoken language. This control allows scientists to conduct experiments with high accuracy and isolate the influence of specific factors on social interaction. The robot behaves in the same way in every session, which is important in research in order to obtain consistent and comparable results. For example, researchers could analyze the participants’ reactions to the same social stimuli. When humanoid robots are used as simulated patients in medical education in the future, a number of aspects related to ethical, practical and social issues should be considered by medical decision makers and the public. Robots offer the advantage of providing consistent and reproducible scenarios, which is important in medical education. The robot can help to improve technical skills, such as diagnostics or examination techniques. The advantages of the technology (such as precision, reproducibility and ethical safety) are clear, but the ethical, social and educational challenges must also be considered.

In addition, a system should be developed to automatically generate robot dynamics. In the future, it is planned to automatically transfer the motion sequences from a human (actor) to the robot using appropriate software. The movements should be detected, interpreted and performed by the robot. There are also further plans to develop structured dialogues for patient communication. A rule-based dialog system should enable complex communication with the robot patient, e.g., for diagnostic findings. The aim is to simulate a real conversation, but limited to a specific topic or scenario. The conversation takes place via spoken language. Another approach is to generate free dialogues for human-like conversations via ChatGPT. In the future, further studies are planned to evaluate the learning success of the students with regard to the training method (actor or robot patient).

## Data Availability

The original contributions presented in the study are included in the article/supplementary material, further inquiries can be directed to the corresponding author.
